# Is dementia research ready for big data approaches?

**DOI:** 10.1186/s12916-015-0367-7

**Published:** 2015-06-22

**Authors:** Martin Hofmann-Apitius

**Affiliations:** Department of Bioinformatics, Fraunhofer Institute for Algorithms and Scientific Computing (SCAI), 53754 Sankt Augustin, Germany

**Keywords:** Big data, Data interoperability, Semantic harmonization, Disease modeling, Data mining, Disease mechanisms

## Abstract

The “big data” paradigm has gained a lot of attention recently, in particular in those areas of biomedicine where we face clear unmet medical needs. Coined as a new paradigm for complex problem solving, big data approaches seem to open promising perspectives in particular for a better understanding of complex diseases such as Alzheimer’s disease and other dementias. In this commentary, we will provide a brief overview on big data principles and the potential they may bring to dementia research, and - most importantly - we will do a reality check in order to provide an answer to the question of whether dementia research is ready for big data approaches.

## Background

According to Wikipedia, *“Big data is a broad term for data sets so large or complex that traditional data processing applications are inadequate. Challenges include analysis, capture, data curation, search, sharing, storage, transfer, visualization, and information privacy. The term often refers simply to the use of predictive analytics or other certain advanced methods to extract value from data, and seldom to a particular size of data set”* [1]. The wide use of the term has contributed to its fuzziness, and the usual IT marketing Newspeak has done its part, too. However, in the original definition by the Gartner group [2] there are three aspects that constitute “big data”; we will use them for the discussion of big data in dementia research. Big data is characterized by:

- **Volume of data.** The “data flood” that we experience in biomedicine, for example, through the enhanced capabilities in the area of genome and transcriptome sequencing technologies, but also in the area of neuroimaging and clinical data
- **Velocity of data.** Usually this “V” is associated with the processing of real-time data analysis (for example, from sensor networks or online trading), but in its core, this concept also covers the heterogeneity of data with respect to time scales.
- **Variety of data.** This is the most interesting aspect of big data when it comes to biomedicine. Linking heterogeneous data is a key step in big data analytics, and shared semantics for metadata annotation play an important role in data integration.

A fourth aspect has recently been added to the big data concepts; it covers the issue of heterogeneous data quality and the need for curation:

- **Veracity of data.** This concept deals with the need for quality assessment, pre-processing, and curation of data. This is of particular importance in biomedicine, where the quality of data and annotations varies in wide ranges.

Given the complexity of neurodegenerative diseases, it is not surprising that we observe a significant heterogeneity of data in that scientific area. Heterogeneity of data concerns their mode (omics data, imaging data, clinical data → ***variety***) as well as their quality (as determined by statistics criteria and with respect to available metadata → ***veracity***). Big data analytics makes use of a wide range of data integration, modeling, and mining strategies in order to understand and predict systems behavior in complex systems. The expectations on complex problem solving capabilities that come with big data approaches are high in the research community working on dementia. As a first big data challenge in Alzheimer’s research, the DREAM challenge has been launched recently [3].

## Promising advances, but a reality check is needed

Big data approaches differ substantially from established biostatistics approaches. Whereas biostatisticians try to keep the number of variables low and put great effort into controlling the experiment, big data analytics accepts as a fundamental premise the heterogeneity of data with respect to both quality and type. Big data approaches try to understand complex systems and try to overcome problems with lacking data by a virtuosic combination of data, knowledge, and imputation of missing values. Interoperability of data and knowledge is key to big data approaches and, therefore, shared semantics (for example, controlled vocabularies; ontologies) play a crucial role not only for metadata annotation, but also for data integration and information extraction procedures. In dementia research, we deal with a wide range of data coming from different levels: omics technologies produce large amounts of quantitative data (such as gene expression data) or qualitative data (for example, single nucleotide polymorphism, SNP, data); neuroimaging generates huge amounts of imaging data that require complex image analysis workflows to extract features from images that can be used in integrative modeling and mining approaches. Dementia research is therefore inherently multi-modal (having different modes of data acquisition) and multi-scalar (ranging from the molecular scale (omics) to the organism (neuroimaging; clinical and cognitive data) and population scales (epidemiological data)).

A reality check of the current situation in dementia with respect to the adoption of big data principles leads us to the DREAM challenge, the first data analytics challenge in the area of research on Alzheimer’s disease [3]. The tasks in this challenge were highly relevant for current Alzheimer’s research, but not too complex and aimed at identifying those features that were informative for the prediction of “cognitive scores 24 months after initial assessment” (subchallenge 1); to “predict the set of cognitively normal individuals whose biomarkers are suggestive of amyloid perturbation” (subchallenge 2); and to “classify individuals into diagnostic groups using MR images” (subchallenge 3).

The data sets made available for the DREAM challenge did not bring along the challenge to harmonize and curate them across different scales or modes of measurement. In that respect, the DREAM challenge on Alzheimer’s disease was not truly a big data challenge. It was rather a statistical quantitative data mining exercise without a decent biological context or knowledge component to integrate. However, attempts to collect and to centrally provision research data in the dementia arena (neuGrid4you [4]; AETIONOMY [5]; Alzheimer’s & Dementia knowledge resource [6]) force us to pay much more attention to the most important V’s: **V(ariety)** and **V(eracity)**. Over the last three years, our team has spent considerable effort on the quality assessment and curation of all publicly available omics data in the area of neurodegenerative diseases. In order to represent the relevant knowledge in a computable form, we and other groups have generated models of disease [7, 8] that represent a good part of the knowledge about Alzheimer’s and Parkinson’s diseases and make this knowledge amenable for computer-based reasoning approaches [9]. Together with curated omics data and additional efforts on clinical data, these models form the basis for the most comprehensive knowledge base on neurodegenerative diseases, the AETIONOMY knowledge base [10]. This knowledge base will support future big data approaches in dementia research by harmonized annotations across heterogeneous data sets and tight integration of disease models, literature-based knowledge, and primary experimental data. Other resources that are currently being built include the AMP AD partnership [11]; a dedicated topic on big data in Alzheimer’s research can be found on the website indicated in Ref. [12].

## Future trends and emerging technologies

**Fig. 1:**
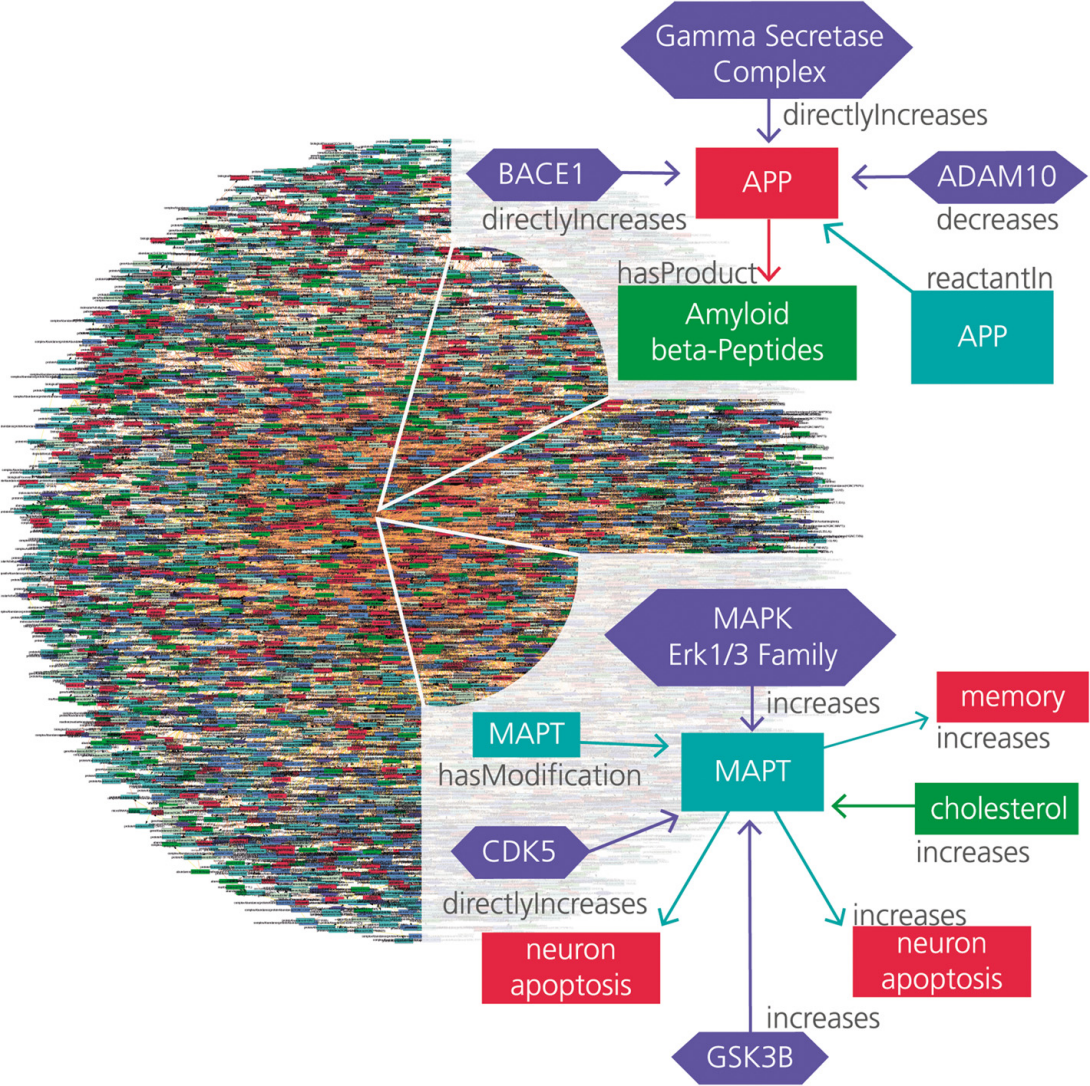
Alzheimer’s disease model encoded in OpenBEL with zoom presentation of the major pathology mechanisms (tau, APP)

A spectrum of currently emerging technologies will add to the **V(olume)** of data relevant for dementia research: next-generation sequencing (NGS) and in particular RNAseq technologies will generate high-quality gene expression data; epigenetics studies in the dementia context will add an entire level of new information. Patient-specific iPS cells will be analyzed simultaneously for gene expression, epigenetics mechanisms, and proteomics (including pathway regulation). More data will also be generated at the clinical level: large observational studies (such as the Rhineland Study conducted by the German National Dementia Research Center [13]) will produce huge amounts of clinical and imaging data. The interpretation of data generated at various different levels and including data that come from either cell and tissue cultures or animal models will require new approaches for integrative data analysis. Big data principles will therefore become even more relevant for integrative modeling and mining and any sort of “systems approach” in dementia research. The computable Alzheimer’s disease model shown in Fig. 1 (a model representing cause-and-effect relationships under disease conditions) captures and represents knowledge about disease processes in Alzheimer’s disease and is ideally suited to support the enhanced interpretation of big data in the Alzheimer’s research area.

## Conclusions

We can expect that, in the near future, data production at all levels - from the omics level to the clinical and population level - will increase with the same rate in dementia research that we can observe in other indication areas. The need for increased interoperability of data (and knowledge!) will simultaneously increase, and substantial effort will be required to cope not only with the rapid growth of data volume, but also with the notorious lack of interoperability of data, information, and knowledge. We will see more ambitious mining scenarios in big data challenges in the future, and there is no doubt: integrative modeling and mining approaches will come, and they will have a strong impact on dementia research.
